# Oxidative Stress Mediates Microcystin-LR-Induced Endoplasmic Reticulum Stress and Autophagy in KK-1 Cells and C57BL/6 Mice Ovaries

**DOI:** 10.3389/fphys.2018.01058

**Published:** 2018-08-06

**Authors:** Haohao Liu, Xiaofeng Zhang, Shenshen Zhang, Hui Huang, Jinxia Wu, Yueqin Wang, Le Yuan, Chuanrui Liu, Xin Zeng, Xuemin Cheng, Donggang Zhuang, Huizhen Zhang

**Affiliations:** Department of Environmental Health, College of Public Health, Zhengzhou University, Zhengzhou, China

**Keywords:** microcystin-leucine arginine (MC-LR), oxidative stress, endoplasmic reticulum stress (ERs), autophagy, *N*-acetyl-l-cysteine (NAC)

## Abstract

Microcystin-leucine arginine (MC-LR) is a cyclic heptapeptide intracellular toxin released by cyanobacteria that exhibits strong reproductive toxicity. However, little is known about its biotoxicity to the female reproductive system. The present study investigates unexplored molecular pathways by which oxidative stress acts on MC-LR-induced endoplasmic reticulum stress (ERs) and autophagy. In the present study, immortalized murine ovarian granular cells (KK-1 cells) were exposed to 8.5, 17, and 34 μg/mL (IC_50_) of MC-LR with or without *N*-acetyl-l-cysteine (NAC, 10 mM) for 24 h, and C57BL/6 mice were treated with 12.5, 25.0, and 40.0 μg/kg⋅bw of MC-LR with or without NAC (200 mg/kg⋅bw) for 14 days. The results revealed that MC-LR could induce cells apoptosis and morphologic changes in ovarian tissues, induce oxidative stress by stimulating the generation of reactive oxygen species (ROS), destroying antioxidant capacity, and subsequently trigger ERs and autophagy by inducing the hyper-expression of ATG12, ATG5, ATG16, EIF2α (phosphorylated at S51), CHOP, XBP1, GRP78, Beclin1, and PERK (Thr980). Furthermore, NAC pretreatment partly inhibited MC-LR-induced ERs and autophagy *via* the PERK/ATG12 and XBP1/Beclin1 pathways. These results suggest that oxidative stress mediated MC-LR-induced ERs and autophagy in KK-1 cells and C57BL/6 mice ovaries. Therefore, oxidative stress plays an important role in female toxicity induced by MC-LR.

## Introduction

Due to the aggravating environmental pollution, including water pollution, the relationship between the decline in human fertility and environmental exposure has attracted worldwide attention. Furthermore, increasing evidences have revealed that microcystin (MC) exposure is closely correlated to reproductive toxicity (Chen et al., 2011, 2016). MC is a family of cyclic heptapeptide intracellular toxins released by cyanobacteria in eutrophication water, and has been regarded as a major health hazard to humans. More than 100 MC variants have been identified (Puddick et al., 2014; Qi et al., 2015; Bouhaddada et al., 2016), and microcystin-leucine arginine (MC-LR) is one of the most common and potent variants (Kaasalainen et al., 2012). In 1998, the World Health Organization established a safety guideline value of 1 μg/L of MC-LR in drinking water (World Health Organization [WHO], 1998). However, the concentration is usually much higher in natural water (13 μg/L) (Amrani et al., 2014). The MC content in the cyanobacteria of water bloom was varies from 0.14 to 13,000 μg/L (Wu et al., 2015). MC is soluble in water, heat-resistant and chemically stable (Merel et al., 2013; Pavagadhi and Balasubramanian, 2013), and it is difficult to remove from nature water used for drinking water supply by existing processing methods. Hence, it is hard to prevent harm when humans and animals are exposed to MC via diet, breathing, and skin contact (Zhang et al., 2009; Zhang D. et al., 2013).

MC-LR can accumulate in several tissues, such as the liver, kidney, and muscle (Lei et al., 2008; Wang et al., 2008). It can also accumulate in the gonads of animals, and transfer from matrix to offspring (Chen et al., 2016). Furthermore, fishers have been found to be positive for serum MC content, which ranged within 0.10–0.64 μg/L (Zhao et al., 2016). Zheng et al. (2017) found that the median serum MC-LR in women was 0.60 μg/L. The toxicity of MC-LR is governed by the irreversible inhibition of protein phosphatase 2A (PP2A) and protein phosphatase 1 (PPl) (Wang L. et al., 2013). Previous studies conducted by the investigators demonstrated that MC-LR could increase reactive oxygen species (ROS) and induce apoptosis in rat Sertoli cells through the mitochondria-mediated signaling pathway (Huang et al., 2016). Although most studies on MC-LR-induced reproductive toxicity have focused on the male reproductive system, the severe toxicity of MC-LR to the female reproduction system should also be given attention. MC-LR can distribute in the ovaries, leading to oxidative stress, cytoskeleton destruction, and gonadal hormone concentration disorder *in vivo*, and inducing the apoptosis of Chinese hamster ovary (CHO) cells by arresting the cell cycle (Gacsi et al., 2009; Chen et al., 2016; Hou et al., 2016). MC-LR has estrogenic potential on fishes and mammalian cells, which influence the normal reproduction of humans, fish, and mammals due to hormonal disorders (Oziol and Bouaicha, 2010; Zhao et al., 2015). MC-LR also could induce disorder of miRNAs and mRNAs in ovarian granulosa cells, which affected the expression of related genes (Li et al., 2017). In addition, the previous studies conducted by the investigators also revealed that endoplasmic reticulum stress (ERs) and autophagy may play a vital role in CHO cell toxicity after MC-LR treatment (Zhang et al., 2016). However, the concrete mechanism remains unknown.

The ovary is vulnerable to oxidative injury, because it is rich in unsaturated lipids. The study conducted by Hou et al. (2014) confirmed that MC-LR induced oxidative stress in zebrafish ovary. Wu et al. (2015) also found MC-LR exposure induced oxidative stress in granulosa cells. Oxidative stress is a stress response induced by excessively high reactive molecules, such as ROS. *N*-acetyl-l-cysteine (NAC) is a vigorous antioxidant that could clear free radicals against oxidative stress by enhancing the generation of glutathione (GSH) *via* deacetylation, in order to generate cysteine after entering the cell (Yarema et al., 2009). Although ROS plays a serviceable role in maintaining cell metabolism and signal transduction, high-level ROS induced by external stimuli can induce oxidative stress, causing ER dysfunction. ER dysfunction can result in a large stack of unfolded proteins or misfolded proteins to ERs, and further lead to unfolded protein response (UPR) (Qu et al., 2013; Zhang et al., 2015; Ryu et al., 2017). UPR is regulated by transmembrane protein sensors, such as PER-like ER kinase (PERK), inositol requiring-1 (IRE1), and activating transcription factor-6 (ATF6), which could combine glucose-regulated protein 78 (GRP78), locating on ER in normal physiological situations. GRP78 can isolate from the transmembrane protein to combine with unfolded protein to activate PERK and X-box binding protein-1 (XBP-1) in ERs and UPR (Gardner and Walter, 2011; Hetz, 2012).

PER-like ER kinase is a sensor protein on the ER membrane, which can regulate protein synthesis to decrease ERs in early UPR. Furthermore, PERK can separate from GRP78, induce the phosphorylation of eIF2α, and modulate ATG5, ATG12, and ATG16, inducing autophagy (Eizirik et al., 2008; B’Chir et al., 2013; Dey et al., 2013). The phosphorylation of eIF2α could also increase the expression of C/EBP homologous protein (CHOP) to induce apoptosis *via* activating transcription factor 4 (ATF4) in an excessive or persistent response to ERs (Rutkowski et al., 2006; Puthalakath et al., 2007; Han et al., 2013; Zhong et al., 2015). XBP-1 is a basic leucine zipper structural protein. It is a marker protein of the ERs and a transcription factor of UPR in ERs, which can adjust protein-folding to minor ERs *via* targeting multiple downstream genes (Iwakoshi et al., 2003; Glimcher, 2010). In addition, XBP-1 can trigger an autophagic signaling pathway through the transcriptional regulation of Beclin1, and induce apoptosis *via* XBP1-IRE1α signaling pathway (Margariti et al., 2013; Song et al., 2013).

To our knowledge, oxidative stress appears to play crucial roles in ERs and autophagy. However, no previous studies have combined ERs and autophagy by ROS mediated in MC-LR-induced reproductive toxicity. Therefore, the aim of the present study was to investigate oxidative stress level and antioxidant ability *in vitro* and *in vivo* following treatment with MC-LR or NAC. Furthermore, the present study will explore the ROS-mediated PERK-EIF2α-ATG12 and XBP1-Beclin1 pathways, and reveal the molecular mechanisms of the protective effects of NAC on MC-LR-induced reproductive toxicity.

## Materials and Methods

### Chemicals

Microcystin-leucine arginine with a purity of >95% was purchased from Beijing Express Technology Co. (Beijing, China). An institutional safety procedure was used to carry out the experiment, according to the textbook of the “*Experimental methods and techniques of Toxicology.*” Dulbecco’s modified eagle medium/nutrient mixture high-glucose (DMEM/high-glucose) and phosphate buffered saline (PBS) were purchased from Hyclone (Logan, UT, United States). Fetal bovine serum (FBS), penicillin–streptomycin and 0.25% trypsin were purchased from GIBCO (Rockville, MD, United States). NAC was purchased from Sigma-Aldrich (St. Louis, MO, United States). The Annexin V-FITC/propidium iodide (PI) apoptosis detection kit, GSH and oxidized glutathione (GSSG) Assay Kit, Total Superoxide Dismutase Assay Kit with WST-8, and Lipid Peroxidation Malondialdehyde (MDA) Assay Kit were purchased from Beyotime Institute of Biotechnology (Shanghai, China). The ROS Assay Kit was purchased from Nanjing Jiancheng Bioengineering Institute (Nanjing, Jiangsu, China). Cell Counting Kit-8 (CCK-8) was purchased from Dojindo Laboratories (Kyushu Island, Japan).

### Cell Culture and Treatments

Immortalized murine ovarian granular KK-1 cells (KK-1 cells), which were obtained from *WT Xu* (College of Food Science and Nutritional Engineering, China Agricultural University) and *HS Luo* (College of Biological Sciences, China Agricultural University) (Li et al., 2014; Li Y. et al., 2015), were grown in DMEM/high-glucose enriched with 10% FBS, 4.0 mM of L-glutamine, 4,500 mg/L of glucose, and 100 U/mL of penicillin/streptomycin. Then, cells were cultured in a humidified CO_2_ chamber at 37°C under normal cell culturing conditions. The MC-LR stock solution was dissolved in PBS to generate 1 mg/mL of stock solution, and this was further diluted with culture medium to the desired concentrations, prior to incubation with KK-1 cells for 24 h.

### Cell Viability Assay

KK-1 cells were plated into a 96-well plate at a density of 2 × 10^5^ cells per mL. When cell density reached up to 80–90%, KK-1 cells were treated with MC-LR at final concentrations of 0, 1, 5, 10, 20, 40, and 60 μg/mL for 24 h, or with NAC at final concentrations of 0, 1, 5, 10, 15, 20, and 40 mM for another 24 h. Each well was washed once with PBS, added with CCK8 reagents (1:10), and incubated at 37°C for 30 min. Optical density was measured using an automated microplate reader (BioTek, Winooski, VT, United States) at 450 nm. Then, the cell viability was calculated, and the IC_50_ of MC-LR or the subsequent experimental concentration of NAC was determined. Cell viability = [(As – Ab)/(Ac – Ab)] × 100%. As: experimental hole absorbance (including medium, cells, CCK8, MC-LR, or RES), Ac: control hole absorbance (including medium, cells, CCK8, non-MC-LR, or RES), Ab: blank hole absorbance (including medium and CCK8, non-cells, non-MC-LR, or RES).

### Apoptosis Assay

The apoptosis rate of KK-1 cells was detected using an Annexin V-fluorescein isothiocyanate/propidium iodide (FITC/PI) Apoptosis Detection Kit *via* flow cytometry. Briefly, cells were seeded into a 6-well plate and exposed to various concentrations of MC-LR (0, 8.5, 17, and 34 μg/mL) with or without NAC (10 mM). After incubation for 24 h, cells were collected, washed twice with cold PBS, and centrifuged at 1,500 × *g* for 5 min. Then, cells were resuspended in 195 μL of binding buffer at concentrations of 5 × 10^5^ cells/mL, and stained with 5 μL of Annexin V-FITC and 10 μL of PI. These cells were kept in the dark at 23°C for 20 min, and subjected to flow cytometry using a FACS Calibur flow cytometer (BD Accuri C6, Franklin Lakes, NJ, United States).

### ROS Assay

*In vitro*, KK-1 cells were treated with MC-LR with or without NAC, and loaded with 10 μM of 2′-7′-dichlorofluorescein diacetate (DCFH-DA) for 30 min at 37°C in the dark. Then, fluorescence was detected using a FACS Calibur flow cytometer (BD Accuri C6, Franklin Lakes, NJ, United States) or laser scanning confocal microscope (Leica, Heidelberg, Germany). *In vivo*, all cells were isolated from fresh ovaries of C57BL/6 mice. The ovaries were washed twice with pre-cold PBS, sheared, and digested with 0.25% trypsin in a shaking water bath at 37°C for 30 min. Then, the homogenate was filtered through a 300-mesh stainless steel filter. Afterward, all cells were collected, washed twice with PBS, and the concentration was adjusted to 1 × 10^7^ cells/mL. Subsequently, cells were loaded with DCFH-DA (10 μM) for 30 min at 37°C in the dark, and fluorescence was detected using an automated microplate reader (BioTek, Winooski, VT, United States).

### SOD and GSH/GSSG Assay

The activity of superoxide dismutase (SOD) was measured using a colorimetric assay kit, according to the WST-8 method. The cellular or ovarian homogenate was centrifuged at 1,500 × *g*/min for 5 min at 4°C, the supernatant was collected, and absorbance was measured at 450 nm using a microplate reader (BioTek, Winooski, VT, United States).

Glutathione and oxidized glutathione content was determined *via* the colorimetric method, according to manufacturer’s instructions. Briefly, the cellular or ovarian homogenate was centrifuged at 10,000 × *g*/min for 10 min at 4°C, the supernatant was collected, GSH working fluid was added, and absorbance was measured at 412 nm using a microplate reader (BioTek, Winooski, VT, United States).

### Western Blotting

Total protein was isolated from the ovary, and KK-1 cells were exposed to various concentrations of MC-LR with or without NAC. Then, the protein content was measured using a BCA Protein Assay Kit (Beyotime, Shanghai, China). Samples that contained 30 μg of protein were separated using SDS-PAGE, and transferred onto a polyvinylidene fluoride (PVDF) membrane (Millipore, Bedford, MA, United States). Then, the membrane was blocked with TBST containing 5% BSA at room temperature for 2 h, and immunoblotted using primary anti-ATG12 (ab155589), anti-ATG5-ATG12 (ab155589), anti-ATG5 (ab108327), anti-ATG16 (ab188642), anti-EIF2α (ab5369), anti-EIF2α (phosphoS51, ab32157), anti-CHOP (ab11419), anti-XBP-1 (ab37152), anti-GRP78 (ab25192), anti LC3 (ab81785) and anti-Beclin1 (ab62557) (Abcam, Cambridge, United Kingdom), and anti-PERK (3192) and anti-PERK (Thr980) (3179) (Cell Signaling Technology, Boston, MA, United States), and anti-MC-LR (MC8C10) (Express Technology CO, Beijing, China). Finally, the membranes were treated with HRP-coupled secondary antibodies (1:5,000, dilution) for 90 min. The protein bands were analyzed using an enhanced chemiluminescence detection kit (Beijing ComWin Biotech, Beijing, China). The intensity of the bands was quantified using the Bio-Rad Quantity One software (Bio-Rad, Hercules, CA, United States). The biological replicates of Western blotting were three times.

### Animal Treatment

Specific pathogen free (SPF) 6-week-old female C57BL/6 mice were obtained from the Beijing Weitong Lihua Experimental Animal Technology Co. Ltd. (Beijing, China) and were fed at the barrier environment animal laboratory of colleague of public health in Zhengzhou University (license number: SYXK (YU) 2012-0007). Mice were fed with standard rodent pellet diet (purchased from the Experimental Animal Center of Henan Province, Zhengzhou, China), provided with water *ad libitum*, and kept on a 12-h light/dark cycle. The experiments on mice were carried out according to the guide for the care and use of the Institutional Animal Care and Use Committee (IACUC) published by the Ministry of Health of the People’s Republic of China. All studies were approved by the Animal Study Committee of the Zhengzhou University. Mice were randomly divided into six groups: control group, NAC group (NAC, 200 mg/kg⋅bw) (Terneus et al., 2008), low-dose group (MC-LR, 12.5 μg/kg⋅bw), medium-dose group (MC-LR, 25 μg/kg⋅bw), high-dose group (MC-LR 40 μg/kg⋅bw), and NAC+MC-LR (40 μg/kg⋅bw) group. Each group had 20 mice. Mice were treated daily with MC-LR or vehicle by intraperitoneal injection for 14 days. Mice in the NAC+MC-LR group were pretreated with NAC for 2 h prior to MC-LR injection. At 24 h after the last injection, the ovaries of mice were excised for analysis.

### Hematoxylin and Eosin (HE) Staining

The ovary was quickly separated from C57BL/6 mice, washed with cold PBS, and fixed in 4% paraformaldehyde overnight. Then, the ovary was equilibrated in a phosphate-buffered 30% sucrose solution, embedded in paraffin, and cut into 6-μm coronal sections. Afterward, the ovary specimens were dewaxed by xylene and alcohol, and stained with hematoxylin and eosin (HE). The morphological changes were observed under a microscope (Nikon Eclipse E100, Japan). Three mice from different group were tested in each group.

### TUNEL Assay

Apoptosis was assessed by terminal deoxynucleotidyl transferase dUTP nick end labeling (TUNEL) assay (Roche, Switzerland), according to manufacturer’s instructions. Briefly, the ovary was quickly separated from C57BL/6 mice, washed with cold PBS, fixed in 4% paraformaldehyde overnight at room temperature, permeabilized with 0.1% Triton X-100, and washed twice. Then, the TdT-labeled nucleotide mix was added to each slide, incubated for 1 h at 37°C and observed using a fluorescent microscope (Olympus, Tokyo, Japan) at 488 nm excitation and 530 nm emission. Image-pro plus 6.0 (Media Cybernetics Inc., Rockville, MD, United States) was used to select the labeled granulosa cells with the green fluorescent nuclei as a unified standard for judging positive cells. Follicles were selected as analysis areas and three whole ovarian sections from different mice were examined.

### MDA Assay

Malondialdehyde was measured using a colorimetric assay kit, according to the reaction of MDA with thiobarbituric acid, in order to produce a red compound. Briefly, the ovary homogenate was centrifuged at 1,600 × *g*/min for 10 min at 4°C, the supernatant was collected, and absorbance was measured at 532 nm using a microplate reader (BioTek, Winooski, VT, United States), according to manufacturer’s instructions.

### Ultrastructure Observation

The ovary was quickly and carefully separated from C57BL/6 mice, washed with cold PBS, and fixed in 2.5% glutaraldehyde for 4 h. Then, the ovary was washed thrice with PBS, dehydrated by alcohol, embedded overnight, and cut into 60-nm coronal sections using an ultramicrotome (Leica, Heidelberg, Germany). Finally, the sections were stained with both uranyl acetate and lead citrate, and the ultrastructure of the ovary was observed using a HT7700 transmission electron microscope (HITACHI, Japan). Three mice from different group were tested in each group.

### Statistical Analysis

Data are expressed as mean ± standard deviation (SD). All statistical analyses were carried out using SPSS 21.0 (SPSS Inc., Chicago, IL, United States). One-way analysis of variance (ANOVA) was used to analyze the significant differences between groups. Student–Newman–Keuls test (SNK) was used for multiple comparisons in variances with homogeneity, and Dunnett T3 test was used for variances without homogeneity. *P* < 0.05 was considered statistically significant.

## Results

### Measurement of Cell Viability and MC-LR Detected in KK-1 Cells and Ovary

**FIGURE 1:**
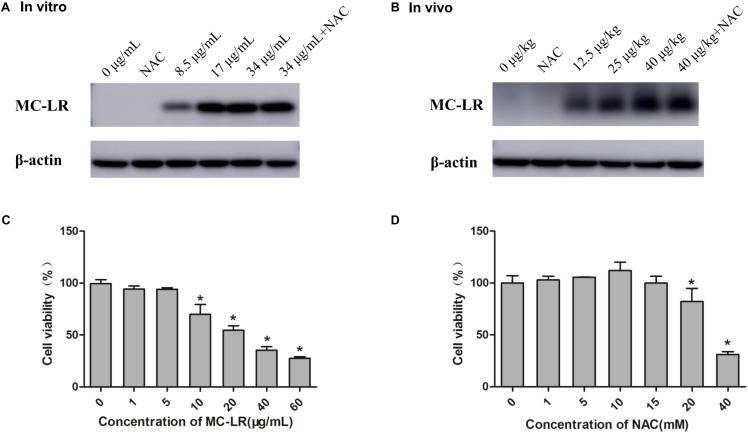
Measurement of cell viability and microcystin-LR (MC-LR) detected in KK-1 cells and ovaries **(A)** MC-LR detected in KK-1 cells exposed to MC-LR with or without *N*-acetyl-l-cysteine (NAC) for 24 h. **(B)** MC-LR detected in C57BL/6 mice ovaries exposed to MC-LR with or without NAC for 14 days. The protein levels of MC-LR in KK-1 cells or mice ovarian tissue were detected by Western blotting. The detected bands (˜39 kD) are adducts of MC-LR-protein phosphatases 1 and 2A (PP1/2A). **(C)** The effect of MC-LR (0–60 μg/mL) on the viability of KK-1 cells for 24 h. **(D)** The effect of NAC (0–40 mM) on the viability of KK-1 cells for 24 h. Data were expressed as mean ± standard deviation (SD, *n* = 3), ^∗^*P* < 0.05 vs. the control group.

Cells or mice were exposed in various concentrations of MC-LR with or without NAC, and then MC-LR was detected using Western blotting. As shown **Figures 1A,B**, no MC-LR band was found in NAC and control group (*In vitro*: 0 μg/mL, *In vivo*: 0 μg/kg). But MC-LR band was found in low-dose group (*In vitro*: 8.5 μg/mL, *In vivo*: 12.5 μg/kg), higher-dose group and NAC+MC-LR group, which indicated that MC-LR could enter into KK-1 cells or ovarian tissue. The CCK8 assay demonstrated the effects of MC-LR or NAC on the viability of KK-1 cells for 24 h. Cell viability gradually decreased with the increase in concentration of MC-LR (1–60 μg/mL). The IC_50_ of MC-LR for KK-1 cells was calculated to 34 μg/mL (**Figure 1C**). Hence, IC_50_, IC_50_/2, and IC_50_/4 were used for the subsequent experiments. According to NAC data of CCK8, cell viability was increased (1–10 mM) but wasn’t statistical significance. Cell viability apparently declined after treatment with NAC (20–40 mM) (**Figure 1D**). Although cell viability was not statistically increased from the concentration of 1 mM to 15 mM, the cell viability after treatment with 10 mM of NAC was the highest. In addition, cell viability (15 mM of NAC) decreased, when compared to 10 mM of NAC. Therefore, 10 mM was used for subsequent experiments.

### The Rescuing Effects of NAC on MC-LR-Induced Redox-Related Indicators Changed in KK-1 Cells and Mice Ovaries

**FIGURE 2:**
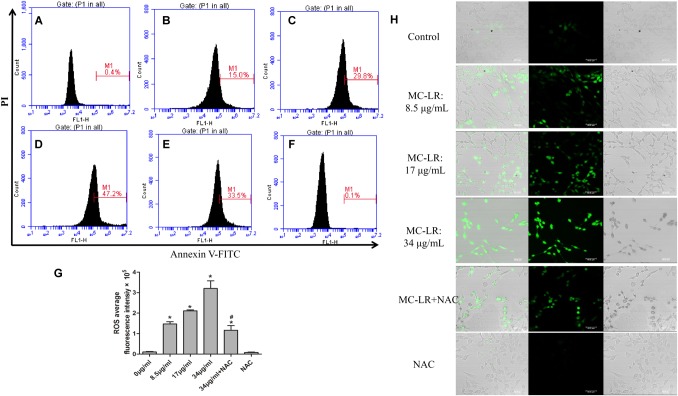
Effect of *N*-acetyl-l-cysteine (NAC) on microcystin-LR (MC-LR)-induced reactive oxygen species (ROS) generation. **(A–G)** DCF fluorescence intensity was detected by DCFH-DA staining and flow cytometry. **(A)** control; **(B)** 8.5 μg/mL; **(C)** 17 μg/mL; **(D)** 34 μg/mL; **(E)** 34 μg/mL+NAC; **(F)** NAC. **(G)** Quantitative analysis of DCF fluorescence in KK-1 cells. Data were expressed as mean ± standard deviation (SD, *n* = 3), ^∗^*P* < 0.05 vs. the control group, ^#^*P* < 0.05 vs. the MC-LR group (34 μg/mL). **(H)** The effect of MC-LR on ROS content in KK-1 cells were observed using a laser confocal microscope (×200), green represent ROS fluorescence intensity, Bar = 40 μm.

**FIGURE 3:**
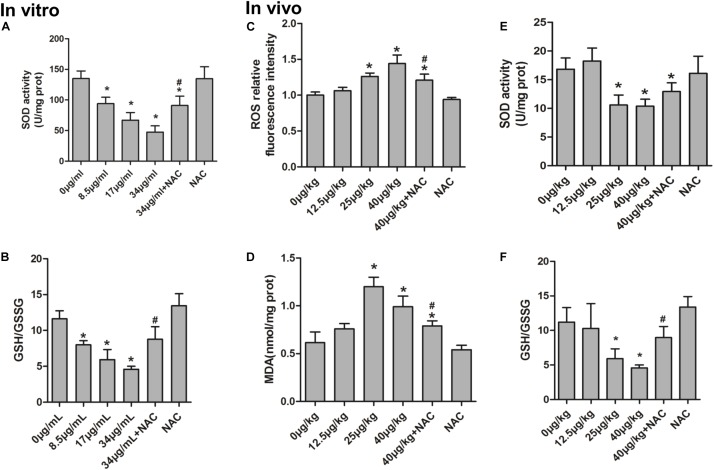
The effect of *N*-acetyl-l-cysteine (NAC) and Microcystin-LR (MC-LR) on redox-related indicators in KK-1 cells and C57BL/6 mice ovarian tissues. **(A)** The activity of superoxide dismutase (SOD) was measured in KK-1 cells. **(B)** glutathione/oxidized glutathione (GSH/GSSG) content was determined *via* the colorimetric method in KK-1 cells. **(C)** The levels of reactive oxygen species (ROS) in mice ovarian tissues were detected using an automated microplate reader *via* monitoring DCF fluorescence intensity. **(D)** Malondialdehyde (MDA) was measured using a colorimetric assay kit in mice ovarian tissues. **(E)** WST-8 method was used to measure the activity of SOD in mice ovarian tissues. **(F)** GSH/GSSG content was determined in mice ovarian tissues. Data were expressed as mean ± standard deviation (SD, *n* = 3), ^∗^*P* < 0.05 vs. the control group, ^#^*P* < 0.05 vs. the MC-LR group (*In vitro*: 34 μg/mL; *In vivo*: 40 μg/kg).

The generation of ROS, which represents the level of oxidative stress, plays a critical role in oxidative stress response and ERs. In order to evaluate the effect of NAC and MC-LR on the intrinsic pathway, KK-1 cells were labeled with DCFH-DA to monitor the intensity of DCF fluorescence *via* flow cytometry and a laser confocal microscope. The results revealed that MC-LR (8.5, 17, and 34 μg/mL) remarkably increased the fluorescence intensity (**Figures 2A–D,G**), and green fluorescence gradually increased with the increase in concentration of MC-LR (**Figure 2H**), when compared to the control group. Moreover, NAC pretreated cells rescued the MC-LR-induced generation of ROS, when compared to the 34 μg/mL of MC-LR group (**Figures 2E–H**). Thus, these results confirm that NAC effectively decreased the MC-LR-induced generation of ROS to mitigate oxidative stress. SOD activity and GSH/GSSG can represent the antioxidant capacity in cells, playing a critical role in antioxidant metabolism. The effect of MC-LR and NAC on antioxidant capacity was also examined. As shown **Figures 3A,B**, SOD activity and GSH/GSSG significantly decreased with the increase in concentration of MC-LR. Compared to the MC-LR group (34 μg/mL), NAC pretreated cells increased the SOD activity and GSH/GSSG. Hence, NAC could resist the MC-LR-induced reduction in antioxidant capacity.

In order to further evaluate the effect of NAC and MC-LR in ovarian tissues, several oxidative products (MDA and ROS) and anti-oxidative factors (SOD and GSH/GSSG) were detected. As shown in **Figures 3C–F**, the relative intensity of fluorescence of MDA and ROS remarkably increased in the medium-dose and high-dose groups, when compared to the control group. Furthermore, NAC pretreated mice weakened the MC-LR-induced generation of ROS and MDA, when compared to the MC-LR (40 μg/kg⋅bw) group (**Figures 3C,D**). Moreover, the present results revealed that SOD activity and GSH/GSSG significantly decreased in the medium-dose and high-dose groups, when compared to the control group. Compared to the MC-LR group (40 μg/kg⋅bw), GSH/GSSG increased in NAC pretreated mice ovaries. However, a slight up-regulation in SOD activity was observed during the NAC pretreatment, but there was no significant difference when compared to the MC-LR (40 μg/kg⋅bw) group (**Figures 3E,F**).

### Effect of NAC and MC-LR on the Protein Levels of ERs and Autophagy in KK-1 Cells

**FIGURE 4:**
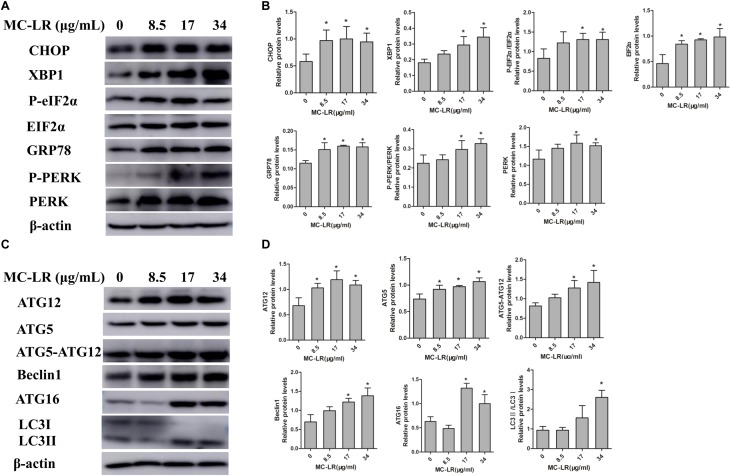
Effect of microcystin-LR (MC-LR) on the protein levels of endoplasmic reticulum stress (ERs) and autophagy in KK-1 cells. **(A)** Western blotting analysis was used to evaluate ERs- related protein expression [PERK, PERK (Thr980), EIF2α (phosphor S51), CHOP, XBP-1, GRP78], and **(C)** autophagy-related protein expression: ATG12, ATG16, ATG5, EIF2α, Beclin1, and LC3. **(B,D)** The expression levels were quantified with Quantity One, and data were represented as mean ± standard deviation (SD, *n* = 3) for each group, ^∗^*P* < 0.05 vs. the control group.

**FIGURE 5:**
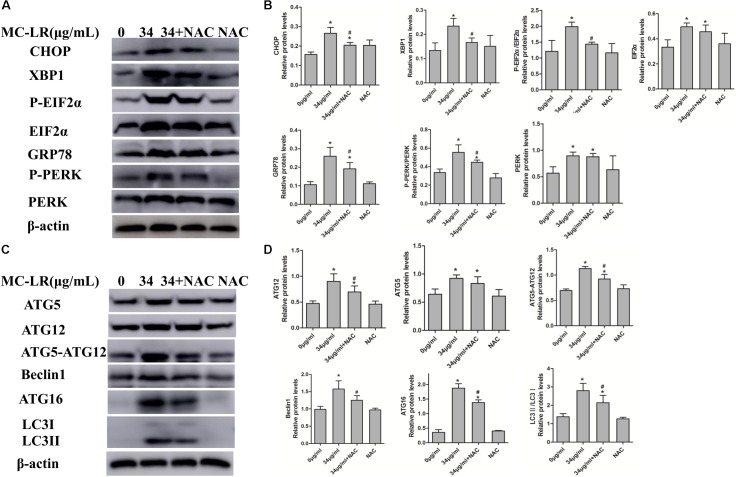
Endoplasmic reticulum stress (ERs)- and autophagy-related protein expression levels in KK-1 cells exposed to microcystin-LR (MC-LR) with or without *N*-acetyl-l-cysteine (NAC) for 24 h. **(A)** Western blotting analysis was used to evaluate ERs- related protein expression [PERK, PERK (Thr980), EIF2α (phosphor S51), CHOP, XBP-1 and GRP78], and **(C)** autophagy-related protein expression: ATG12, ATG16, ATG5, EIF2α, Beclin1, and LC3. **(B,D)** The expression levels were quantified with Quantity One, and data were represented as mean ± standard deviation (SD, *n* = 3) for each group, ^∗^*P* < 0.05 vs. the control group, ^#^*P* < 0.05 vs. the MC-LR group (34 μg/mL).

In order to examine the effect of MC-LR on the protein levels of ERs and autophagy, cells were exposed to MC-LR (8.5, 17, and 34 μg/mL) for 24 h, and the protein expression of PERK/ATG12 and XBP-1/Beclin1 was tested by Western blotting. MC-LR exposure caused a significant increase in the expression of CHOP, GRP78, ATG5, and ATG12 with the increase in concentration of MC-LR. Furthermore, XBP-1, P-EIF2α (P-EIF2α/EIF2α), P-PERK (P-PERK/PERK), Beclin1, ATG16 and ATG5-ATG12 expression were significantly increased only in the 17 μg/mL and 34 μg/mL MC-LR groups. The expression of LC3II/LC3I was significantly increased only in the 34 μg/mL MC-LR group (**Figure 4**). Collectively, these results suggest that MC-LR could induce KK-1 cell ERs and autophagy *via* the PERK/ATG12 and XBP1/Beclin1 pathways. In order to further investigate the effect of NAC on MC-LR-induced ERs and autophagy, cells were pretreated with NAC (10 mM) for 2 h, and followed exposed to MC-LR (34 μg/mL). As shown in **Figure 5**, the expression of XBP1, CHOP, GRP78, P-EIF2α, P-PERK, Beclin1, ATG12, ATG16, LC3II/LC3I, and ATG5-ATG12 was significantly decreased, when compared to the MC-LR group, but there was no significant difference in ATG5, EIF2α and PERK expression (**Figure 5**).

### Effect of NAC on MC-LR-Induced Pathological Change in the Ovaries of C57BL/6 Mice

**FIGURE 6:**
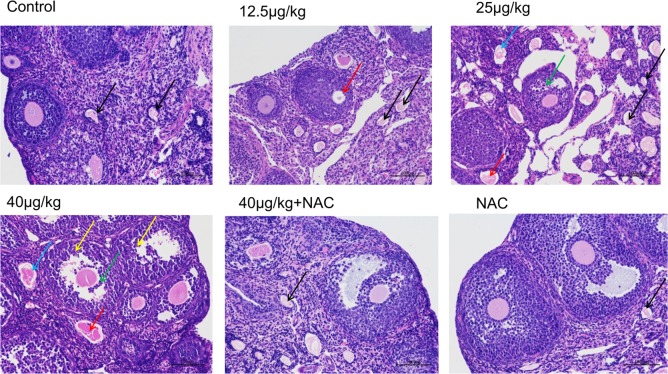
The morphologic changes of ovaries exposed to microcystin-LR (MC-LR) with or without *N*-acetyl-l-cysteine (NAC) (×200). Hematoxylin and eosin (HE) staining with light microscopy was performed to evaluate the effects of MC-LR and NAC on the ovarian histomorphology of C57BL/6 mice. Blue arrow: zona pellucida collapse, green arrow: granulosa cell apoptosis and necrosis, red arrow: oocyte autolysis atrophy, black arrow: atresia follicles, yellow arrow: neutrophil. Bar = 100 μm.

Hematoxylin and eosin staining and a light microscope were used to evaluate the effects of MC-LR and NAC on the ovarian histomorphology of C57BL/6 mice. As shown in **Figure 6**, cells arranged in neat rows and had no pathological changes in control and NAC monotherapy group. A slight oocyte autolysis (red arrow) was found after mice exposure to 12.5 μg/kg⋅bw of MC-LR. Moreover, 25 μg/kg⋅bw of MC-LR induced the granulosa cells to loose arrangement, and caused granulosa cell apoptosis and necrosis (green arrow), oocyte autolysis atrophy (red arrow) and zona pellucida collapse (blue arrow). Moreover, no healthy follicles were found in this group. This kind of effect was more obvious after exposure to higher MC-LR (40 μg/kg⋅bw). In addition, neutrophils (yellow arrow) were found in the high-dose group (40 μg/kg⋅bw). In the NAC+MC-LR (40 μg/kg⋅bw) group, although the ovarian histomorphology showed the follicle transformation into atresia follicles and the loose arrangement of granulosa cells, pathological damage was more slight, when compared to that in mice solely treated with MC-LR (40 μg/kg⋅bw, **Figure 6**).

### Ultrastructural Observations of Ovarian Granulosa Cells in Mice Ovaries

**FIGURE 7:**
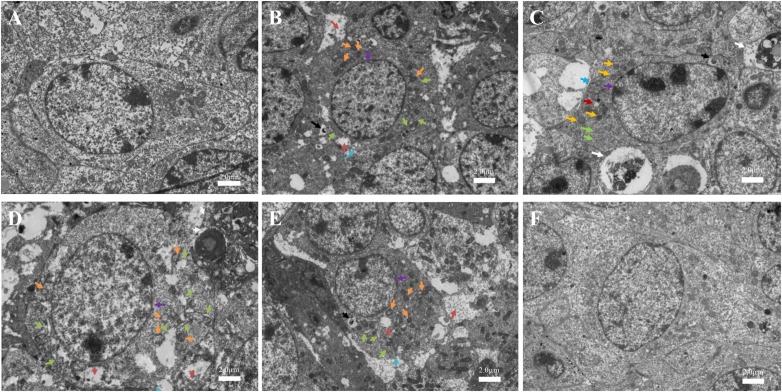
Toxic effect on the ovarian ultrastructures of mice treated with microcystin-LR (MC-LR) with or without *N*-acetyl-l-cysteine (NAC) (×1,500). **(A)** Control; **(B)** 12.5 μg/kg⋅bw; **(C)** 25 μg/kg⋅bw; **(D)** 40 μg/kg⋅bw; **(E)** NAC + 40 μg/kg⋅bw; **(F)** NAC. Rough endoplasmic reticulum (ER) dilation (green arrow), autophagolysosome (black arrow), intracellular edema vacuoles (bronzing arrow), incomplete cell membrane (blue arrow), nucleus (purple arrow), swollen mitochondria (orange arrow), and the apoptosis body (white arrow); Bar = 2.0 μm.

**Figures 7A,F** shows the normal ovarian granulosa cells of C57BL/6 mice in the control and NAC group. Ovarian granulosa cells had intact plasmalemma and normal ER. The mitochondria were ovoid, and their cristae were clear. In the low-dose group (MC-LR, 12.5 μg/kg⋅bw), ovarian granulosa cells in ovary had a rough ER dilation (green arrow), autophagolysosomes (black arrow), intracellular edema vacuoles (bronzing arrow), and incomplete cell membrane (blue arrow). Furthermore, the mitochondria were swollen, and their cristae disappeared (orange arrow, **Figure 7B**). In the medium-dose group, in addition to more damaged mitochondria and ER, apoptosis (white arrow) were also found (**Figure 7C**). These effects were more pronounced after mice were treated with MC-LR (40 μg/kg⋅bw), but autophagolysosome was not observed (**Figure 7D**). When mice were exposed to MC-LR (40 μg/kg⋅bw) with NAC for 14 days, similar dilation of the ER and swelling of the mitochondria were observed. However, slight damage was found, when compared to MC-LR (40 μg/kg⋅bw)-treated mice (**Figure 7E**).

### Protective Effect of NAC on MC-LR-Induced Cell Apoptosis

**FIGURE 8:**
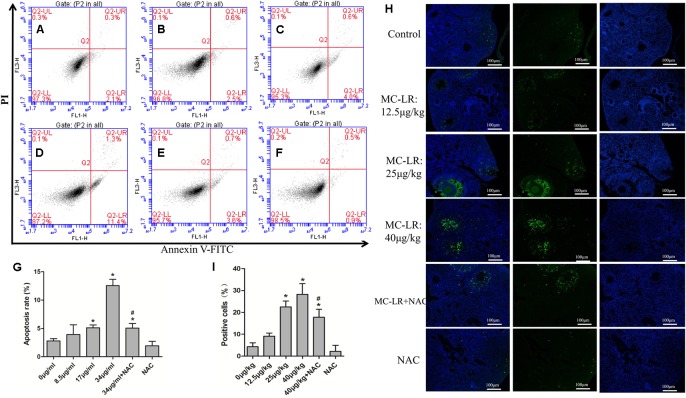
Protective effect of *N*-acetyl-l-cysteine (NAC) on microcystin-LR (MC-LR)-induced ovarian granulosa cell apoptosis. **(A–F)** KK-1 cell apoptosis rate (including early apoptotic cells and late stage apoptotic cells) was detected by flow cytometry *via* Annexin V-FITC/PI apoptosis detection kits. Cells were exposed to various concentrations of MC-LR with or without NAC for 24 h. **(A)** Control; **(B)** 8.5 μg/mL; **(C)** 17 μg/mL; **(D)** 34 μg/mL; **(E)** NAC+34 μg/mL; **(F)** NAC. Q2-LL represents normal cells, Q2-LR represents early apoptotic cells, Q2-UR represents late stage apoptotic cells, and Q2-UL represents necrotic cells. **(H)** Effect of NAC on MC-LR-induced ovarian granulosa cell apoptosis in C57BL/6 mice ovaries (×200). TUNEL assay was used to monitor the apoptosis rate of ovarian granulosa cells in C57BL/6 mice exposed to MC-LR with or without NAC; Green, TUNEL-positive cells; blue, nuclear. Bar = 100 μm. **(G,I)** Data were expressed as mean ± standard deviation (SD, *n* = 3), ^∗^*P* < 0.05 vs. the control group, ^#^*P* < 0.05 vs. the MC-LR group (*In vitro*: 34 μg/mL; *In vivo*: 40 μg/kg).

*In vitro*, the KK-1 cell apoptosis rate was detected by flow cytometry *via* Annexin V-FITC/PI apoptosis detection kits. The apoptosis rate significantly increased in cells exposed to high conditions of MC-LR (17 and 34 μg/mL). However, compared to MC-LR treatment alone, the apoptosis rate of the group pre-treated with NAC (10 mM) remarkably decreased (**Figures 8A–G**). *In vivo*, in order to further evaluate the effect of NAC on MC-LR-induced ovarian granulosa cell apoptosis, the apoptosis rate of ovarian granulosa cells obtained from mice were tested by TUNEL assay *via* confocal microscopy. As shown in **Figures 8H,I**, the expression of ovarian granulosa cell apoptosis (TUNEL-positive) significantly increased in the medium-dose (MC-LR, 25 μg/kg⋅bw) and high-dose (MC-LR, 40 μg/kg⋅bw) groups, when compared to the control group. Interestingly, mice pretreated with NAC for 2 h, followed by MC-LR injection, dramatically alleviated the cell apoptosis rate, when compared to the MC-LR (40 μg/kg⋅bw) group.

### NAC Mediates PERK/ATG12-Related and XBP1/Beclin1-Related Protein Expression in Ovarian Tissues

**FIGURE 9:**
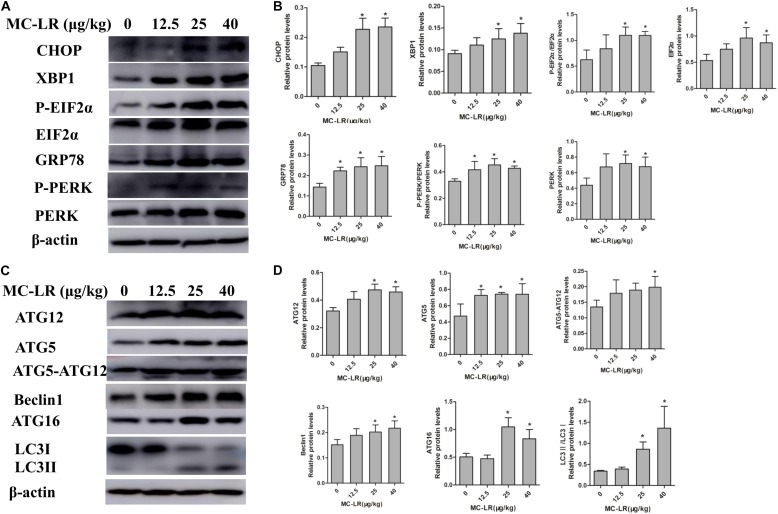
Western blotting of endoplasmic reticulum stress (ERs)-related and autophagy-related proteins in ovarian tissues of mice exposed to microcystin-LR (MC-LR). **(A)** Western blotting analysis was used to evaluate ERs- related protein expression [PERK, PERK (Thr980), EIF2α (phosphor S51), CHOP, XBP-1 and GRP78], and **(C)** autophagy-related protein expression: ATG12, ATG16, ATG5, EIF2α, Beclin1, and LC3 **(B,D)** The expression levels were quantified with Quantity One, and data were presented as mean ± SD for each group, *n* = 3, ^∗^*P* < 0.05 vs. the control group.

**FIGURE 10:**
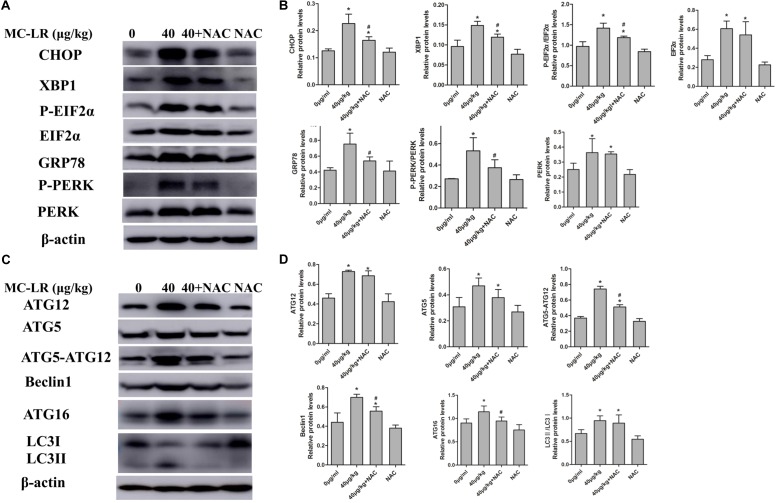
Endoplasmic reticulum stress (ERs)- and autophagy-related protein expression in ovarian tissues of mice exposed to microcystin-LR (MC-LR) with or without *N*-acetyl-l-cysteine (NAC). **(A)** Western blotting analysis was used to evaluate ERs- related protein expression [PERK, PERK (Thr980), EIF2α (phosphor S51), CHOP, XBP-1, GRP78], and **(C)** autophagy-related protein expression: ATG12, ATG16, ATG5, EIF2α, Beclin1, and LC3 **(B,D)** The expression levels were quantified with Quantity One, and data were presented as mean ± standard deviation (SD) for each group, ^∗^*P* < 0.05 vs. the control group, *n* = 3, ^#^*P* < 0.05 vs. the MC-LR group (40 μg/kg).

In order to examine the effect of NAC on MC-LR-induced ERs and autophagy in ovarian tissues, the protein expression of ATG5, EIF2α (phosphor S51), CHOP, XBP-1, GRP78, Beclin1, PERK (Thr980) ATG16, LC3II/LC3I and ATG12 were detected by Western blotting. As shown in **Figure 9**, MC-LR induced the PERK/ATG12-related and XBP-1/Beclin1-related protein expression, when compared to the control group. Furthermore, NAC pretreatment suppressed the MC-LR-induced PERK/ATG12-related and XBP-1/Beclin1-related protein expression, except for EIF2α, PERK, ATG5, ATG12, and LC3II/LC3I which were found to be similar to that in the MC-LR group (40 μg/kg⋅bw, **Figure 10**). It was hypothesized that the protection effect of NAC against MC-LR-induced ERs and autophagy might attribute to the inhibition of the PERK/ATG12 and XBP-1/Beclin1 pathways.

## Discussion

Environmental exposure to MC-LR can cause a variety of serious health problems, including reproductive toxicity, with a resultant decline in fertility (Chen et al., 2011, 2016). Most researches on the reproductive toxicity of MC-LR have focus on the male reproductive system. Recently, some studies have demonstrated that MC-LR could distribute in the ovary, inducing female reproductive toxicity, and the female gender appears to be more sensitive than the male gender (Gacsi et al., 2009; Qiao et al., 2013; Hou et al., 2014; Wu et al., 2014). In the present study, a combination of *in vitro* and *in vivo* studies was first conducted to investigate the mechanistic basis of the toxic effects of MC-LR on the female reproduction system.

Ovarian granulosa cells are one of the most primary functional cells in the follicle, which maintain a microenvironment conducive to oocyte growth and maturation *via* the secretion of steroid hormone, follicle-stimulating hormone, luteinizing hormone, and cytokine. It plays a vital role in modulating ovarian local circumstance, and is a superb model to study MC-LR-induced female reproductive toxicity. In the present study, MC-LR was found to enter into KK-1 cells or ovarian tissues treated with various concentrations of MC-LR, which were similar to discovery of other investigators (Wu et al., 2014, 2015). In addition, oocyte atresia and degenerated vitellogenic oocytes was found to be elevated in MC-LR-treated female zebrafish (Hou et al., 2014). MC-LR could also reduce the number of primordial follicles in female Balb/c mice (Wu et al., 2014). In the present study, MC-LR-induced pathological change was serious. MC-LR induced granulosa cells to lose their arrangement. Granulosa cell apoptosis and necrosis, oocyte autolysis atrophy, and zona pellucida collapse also were found in the MC-LR-treated groups. However, mice pretreated with NAC for 2 h, followed by MC-LR injection, dramatically alleviated the pathological change, when compared to the MC-LR (40 μg/kg⋅bw) group. It was speculated that NAC ameliorated the MC-LR-induced pathological change. MC-LR also increased the ovarian granulosa cell apoptosis rate *in vivo* and *in vitro*. However, NAC pretreatment suppressed the cell apoptosis. Furthermore, MC-LR also induced ultrastructural lesions in the present study. MC-LR treatment resulted in granulosa cell membrane breakage, mitochondria and ER swelling, which caused the occurrence of autolysosome. The present research found that the ultrastructural lesions were more serious than sub-chronic exposure MC-LR-induced medaka fish ovary damage (Trinchet et al., 2011). Interestingly, NAC pretreatment revealed a slight damaged, when compared to MC-LR (40 μg/kg⋅bw) treated mice. Hence, NAC could protect MC-LR-induced ultrastructural damage.

The ovary is vulnerable to oxidative injury, because it is rich in unsaturated lipids. Many previous studies have proven that MC-LR induced oxidative stress, which appeared to be the first step in inducing reproductive toxicity. MC-LR increased the levels of MDA and ROS in testicular tissue and male germ cells. However, the activity of SOD increased *in vivo* and decreased *in vitro* (Li and Han, 2012; Chen et al., 2016). Concurrently, MC-LR increased the MDA and SOD activity, and reduced the level of GSH in zebrafish ovary (Hou et al., 2014). In addition, it was demonstrated that MC-LR could increase the generation of MDA and decrease SOD activity in granulosa cells (Wu et al., 2015). The present study found that GSH/GSSG was inhibited, and the levels of ROS and MDA increased in both MC-LR-treated KK-1 cells and mice ovary. Interestingly, MC-LR induced the increase in SOD activity *in vivo* and the decrease in SOD activity *in vitro*, while a slight upregulation in SOD activity was found in the low-dose group, but the difference was not statistically significant. The dramatic downregulation of SOD activity in the medium-dose and high-dose groups *in vivo* were similar to MC-LR-induced hepatotoxicity (Wang J. et al., 2013). These results indicate that there might be a decompensation reaction, and antioxidant defense system was destroyed, accompanied by the severe pathological lesion of the ovarian tissue which was evaluated by HE staining and ultrastructure observation. NAC is an effective antioxidant (Yarema et al., 2009), which could react against MC-LR-induced oxidative stress in CHO cells and Sertoli cells, as reported by previous studies conducted by the investigators (Xue et al., 2015; Huang et al., 2016). Furthermore, antioxidant κ-Selenocarrageenan could significantly ameliorate the hepatic damage induced by MC-LR, including oxidative damage and ERs (Wang J. et al., 2013). The present results revealed that ROS levels in the NAC+MC-LR group were lower than that in the MC-LR group, both *in vitro* and *in vivo*. These results indicate that NAC suppressed the oxidative stress *via* clearing ROS. In addition, there was a remarkable recovery in SOD activity (expect ovarian tissue) and GSH/GSSG in the NAC-pretreated group, both *in vitro* and *in vivo*, indicating that NAC could also dramatically improve MC-LR-induced oxidative stress by recovering antioxidant capacity.

Microcystin-leucine arginine could induce oxidative stress, which has generally been considered as an initiating factor of the toxicity of MC-LR in the liver, gonadal and nervous system (Chen et al., 2016; Hu et al., 2016; Valerio et al., 2016). However, there have been no reports on the combined effect of oxidative stress and ERs on MC-LR-induced reproductive toxicity. Emodin could trigger ERs and increase the expression of GRP78, XBP-1, and CHOP, while ROS scavenger NAC almost completely blocked emodin-induced ERs and decreased the expression of GRP78, XBP-1, and CHOP (Qu et al., 2013). ROS also mediated ERs in cigarette-exposed human bronchial epithelial cells (Tagawa et al., 2008). A study found that antioxidant κ-Selenocarrageenan suppressed oxidative stress and mediated the expression of P-EIF2α to ameliorate ERs induced by MC-LR (Wang J. et al., 2013). Zhang H. et al. (2013) revealed that MC-LR induced oxidative stress and ERs in *Rana nigromaculata* testes, and they speculated that oxidative stress occurred upstream of ERs. The present study first demonstrated that MC-LR induced oxidative stress in C57BL/6 mice ovaries and KK-1 cells, which occurred upstream of ERs. Furthermore, the present study revealed that MC-LR potently increased the expression of ER-related proteins, including XBP-1, P-PERK, P-EIF2α, GRP78 and CHOP, while ROS scavenger NAC almost completely blocked MC-LR-induced ERs. ROS-mediated ERs can also induce autophagy *via* ER-associated pathways (Li L. et al., 2015). Saxifragifolin D could induce ERs and autophagy *via* the activation of Beclin1, XBP-1, GRP78, and CHOP in breast cancer cells, while NAC pretreatment blocked autophagy through ROS-dependent ERs (Shi J.M. et al., 2013). NAC could also block both ERs and autophagy induced by oxidant stress-mediated aldosterone/mineralocorticoid receptor-triggered CHOP-dependent podocyte injury (Yuan et al., 2015). Chang et al. (2017) found that the P-eIF2α/ATF4 pathway is involved in the ROS-mediated selection of autophagy in glucose-deprived nucleus pulposus cells. Moreover, Trichokonin VI treatment induced ROS accumulation, which resulted in the subsequent disposal of damaged mitochondria within the autophagosomes *via* Atg5-mediated autophagy (Shi M. et al., 2013). The present study found that the levels of autophagy-related proteins were enhanced, when compared to the control group, in both ovarian tissues and KK-1 cells. In addition, the protein levels of LC3II/LC3I (expect ovarian tissue), ATG12 (expect ovarian tissue), ATG5-ATG12, ATG16 and Beclin1 were significantly suppressed by NAC pretreatment in both mice ovaries and KK-1 cells. It was suggested that oxidative stress mediated MC-LR-induced autophagy.

PERK could separate from GRP78 and be activated to induce the phosphorylation of eIF2α and modulate ATG5, ATG12, and ATG16, leading to autophagy (Harding et al., 1999; Eizirik et al., 2008; B’Chir et al., 2013; Dey et al., 2013). Heat-induced ROS could accelerate autophagy *via* the PERK/eIF2α pathway in the lungs of male rats and 16HBE140 cells (Dong et al., 2017). The present study also found that treatment with MC-LR increased the expression of P-PERK, P-EIF2α, ATG5, ATG12, ATG16, and ATG5-ATG12 *in vivo* and *in vitro*. P-PERK, P-EIF2α, ATG16, and ATG5-ATG12 protein levels in the NAC+MC-LR group were lower than that in the MC-LR group, both *in vitro* and *in vivo*. These results indicate that oxidative stress induces ERs and autophagy through the PERK/ATG12 pathway. The phosphorylation of eIF2α could also increase the expression of CHOP, which induces apoptosis *via* ATF4 in an excessive or persistent response to ERs (Rutkowski et al., 2006; Puthalakath et al., 2007; Han et al., 2013; Zhong et al., 2015). In the present study, NAC pretreatment reduced the expression of CHOP, when compared to the MC-LR group, and attenuated MC-LR-induced ovarian granulosa cell apoptosis, both *in vitro* and *in vivo*, suggesting that oxidative stress mediates the MC-LR-induced apoptosis of granulosa cells. However, apoptosis is regulated not only through the ER pathway, but also through the mitochondrial pathway. ROS could mediate MC-LR-induced apoptosis *via* the mitochondrial caspase-dependent pathway in rat Sertoli cells, as reported in a previous study conducted by the investigators (Huang et al., 2016). Hence, more studies and analyses needs to be performed on MC-LR-induced apoptosis in the ER pathway and mitochondrial pathway mediated by ROS in the female reproductive system. XBP-1 has a significant role in ERs, and can adjust protein-folding to minor ERs *via* targeting multiple downstream genes (Iwakoshi et al., 2003; Glimcher, 2010). In addition, XBP-1 can trigger an autophagic signal pathway through the transcriptional regulation of Beclin1, and induce apoptosis *via* iterations in the XBP1-IRE1α signaling pathway (Margariti et al., 2013; Sano and Reed, 2013; Song et al., 2013). Interestingly, the present study also revealed that the ovarian granulosa cell apoptosis rate and the expression of XBP-1, Beclin1 and CHOP increased, when compared to the control group, while NAC pretreatment reduced ovarian granulosa cell apoptosis and the expression of XBP-1, Beclin1 and CHOP, when compared to the MC-LR group, both *in vivo* and *in vitro*. It is noteworthy that these results explicitly demonstrate that oxidative stress mediates MC-LR-induced ERs and autophagy in KK-1 cells and C57BL/6 mice ovaries.

## Conclusion

The present study revealed that oxidative stress is essential for MC-LR-induced ERs and autophagy in KK-1 cells and C57BL/6 mice ovaries. MC-LR could cause cell apoptosis, ovarian tissue pathology, and induce oxidative stress *via* stimulating the generation of ROS and MDA. These results also demonstrated that MC-LR destroyed the antioxidant capacity, and subsequently triggered ERs and autophagy by inducing the hyper-expression of ATG16, ATG12, ATG5, EIF2α (phosphoS51), CHOP, XBP-1, GRP78, LC3II/LC3I, Beclin1, and PERK (Thr980). Furthermore, NAC pretreatment partly inhibited MC-LR-induced ERs and autophagy *via* the PERK/ATG12 and XBP-1/Beclin1 pathways. Collectively, these results suggest that oxidative stress mediated MC-LR-induced ERs and autophagy both in KK-1 cells and C57BL/6 mice ovaries. These findings provide new insights and mechanism targets for MC-LR-induced female reproduction toxicity.

## Author Contributions

HL worked on the study design, data interpretation, manuscript preparation, and literature search. XZh worked on data collection, data interpretation, and manuscript preparation. SZ worked on data collection, data interpretation, literature search, and manuscript preparation. HH, JW, YW, LY, CL, and XZe worked on data collection and literature search. XC and DZ worked on the literature search. HZ worked on the study design, data interpretation, manuscript preparation, and funds collection.

## Conflict of Interest Statement

The authors declare that the research was conducted in the absence of any commercial or financial relationships that could be construed as a potential conflict of interest.
